# Domain-specific physical activity, sedentary behavior, subjective health, and health-related quality of life among older adults

**DOI:** 10.1186/s12955-023-02136-8

**Published:** 2023-05-29

**Authors:** Jihee Min, Jae Seung Chang, In Deok Kong

**Affiliations:** 1 Department of Convergence Medicine, Yonsei University Wonju College of Medicine, Wonju, Republic of Korea; 2 Yonsei Institute of Sports Science and Exercise Medicine, Wonju, Republic of Korea; 3 National Cancer Survivorship Center, National Cancer Control Institute, National Cancer Center, Goyang-si, Republic of Korea; 4 Department of Sports Science, Hannam University, Daejeon, Republic of Korea

**Keywords:** Domain-specific physical activity, Sedentary behavior, EQ-5D, Subjective health

## Abstract

**Purpose:**

This study aims to investigate the association between domain-specific physical activity (PA), sedentary behavior, subjective health perception, and health-related quality of life (HR-QoL) in Korean adults aged ≥ 65 years.

**Methods:**

This cross-sectional study analyzed 6,004 older adults from the Korean National Health and Nutrition Examination Survey 2017–2020. PA and sedentary behavior were measured using a global PA questionnaire, and HR-QoL was assessed using the EuroQol-5 Dimension (EQ-5D, three-level version). Multiple logistic regression was used to estimate the odds ratios (ORs) and 95% confidence intervals (CIs) after adjusting for confounding parameters.

**Results:**

Older adults who were physically active at work showed a negative association with subjectively good health and HR-QoL, whereas those physically active in transport or leisure time showed a positive association with subjectively good health and HR-QoL. Older adults highly engaged in sedentary behavior showed a worse perception of health and HR-QoL. Compared to high sedentary behavior and physical activity during leisure time or transport, the EQ-5D index was higher than that of their counterparts.

**Conclusion:**

Both domain-specific PA and sedentary behavior were significantly associated with older adults’ perception of health and HR-QoL. Interventions are needed to improve HR-QoL by reducing sedentary behavior and encouraging physical activity in transportation or leisure time among adults aged 65 years and above.

**Supplementary Information:**

The online version contains supplementary material available at 10.1186/s12955-023-02136-8.

## Introduction

Advanced medicine and economic development have extended life expectancy and, along with a decrease in fertility rates, have increased the aging population worldwide [1]. Korea is also facing a huge challenge due to its aging population. In 2017, South Korea became an aged society, with more than 14% of people aged 65 years or older, and is predicted to become a super-aged society by 2025 [2]. An increasingly older population not only causes an increase in medical costs due to physical and psychological aging and disease but also raises the cost of social and economic support [3, 4]. Thus, improving quality of life, represented by the physical and psychological health of older adults, through active aging is becoming an increasingly prominent public health issue [5, 6].

Subjective health perception is one of the often-used tools in an overall measure of both physical and psychological health [7, 8]. Previous studies have reported that those with poor subjective health perception had a higher risk of mortality than those with good subjective health perception [9, 10]. It may be vital to improve subjective health perception to prolong the healthy lifespan of older adults.

Physical activity (PA), along with other lifestyle modifications, is one of the most effective ways to prevent aging-related non-communicable diseases [11–13]. A recent study on older people indicates that physical activity helps to maintain the functional capacities and health of older adults, leading to better life satisfaction [14]. In addition to insufficient PA, highly sedentary behavior may also pose a significant health risk [15, 16]. Increased activity and reduced sedentary behavior in older adults reportedly prevent cognitive and physical functional attenuation, alleviate diverse chronic conditions and symptoms related to aging, and might prevent or even reverse frailty [17–19]. This ultimately leads to improvements in the quality of life of older adults and encourages active aging.

However, most previous studies have focused on the total amount or intensity of PA. Thus, little is known about whether domain-specific PA, such as PA at work, during transportation, and during leisure time, is related to subjective health perception and health-related quality of life (HR-QoL) in older adults. Additionally, previous studies concluded that socio-environmental and contextual levels, such as gender, education, and income, may influence the motives and variables that affect PA level[20, 21]. However, there is still a lack of evidence regarding domain-specific PA. Furthermore, the joint association between domain-specific PA, sedentary behavior, subjective health, and HR-QoL in older adults has not been evaluated.

Therefore, the purpose of the current study was to investigate the association between domain-specific PA, sedentary behavior, subjective health perception, and HR-QoL among Korean individuals aged 65 years or above.

## Methods

### Study participants

**Fig. 1:**
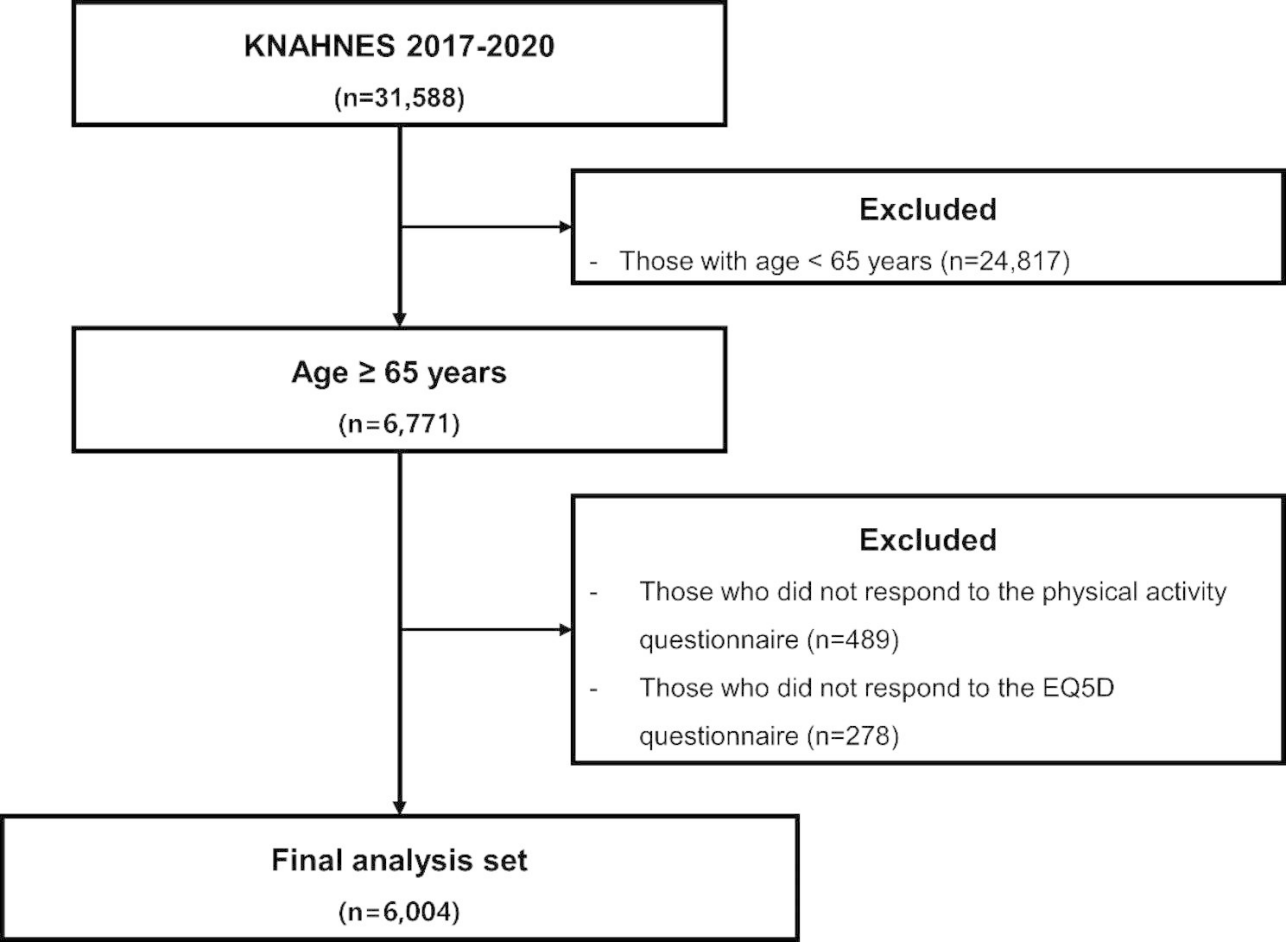
Flow diagram

The secondary data analysis was based on the Korean National Health and Nutrition Examination Survey (KNHANNES) data from 2017 to 2020. The KNHANNES is a nationwide cross-sectional survey conducted every year since 2007 in Korea [22]. Among 31,588 participants, 6,004 were included in the final analysis after excluding those younger than 65 years, with no data on PA, subjective health, and health-related quality of life (Fig. 1). All participants provided informed consent, and this study was approved by the Korea Centers for Disease Control and Prevention Institutional Review Board.

### Measurements

PA level was assessed using the Global Physical Activity Questionnaire (GPAQ). The GPAQ consists of 16 items that collect information about the amount of PA in three domains (leisure time, transport, and work) as well as sedentary behavior. PA in leisure time and work captures intensity (i.e., vigorous, moderate), frequency, and duration. The transport-related PA asked participants to respond to frequency and duration in a typical week. Total PA was calculated by summing the minutes spent per week in each domain. Sedentary behavior consisted of one question: ‘How much do you usually spend sitting or reclining on a typical day?’ and responded to hours and minutes. The validity and reliability of the Korean GPAQ have been evaluated previously [23].

The subjective health state was measured using a single item asking respondents to rate ‘How do you feel about your health in daily life?’. The participants responded on a five-point Likert scale (1 = very healthy to 5 = very unhealthy). Dichotomous variables were defined for a subjective health index as positive vs. neutral/non-positive. HR-QoL was measured using the three-level version of the five-dimension EroQol scale questionnaire (EQ-5D-3L). The EQ-5D-3L comprises the following five dimensions: mobility, self-care, usual activities, pain/discomfort, and anxiety/depression. The response options were scaled to three levels: no problems, some problems, and extreme [24]. We defined dichotomous variables for each EQ-5D-3L dimension according to the presence or absence of any grade of problems (i.e., no problem vs. some/extreme problems).

### Covariates

In the adjusted model, we included covariates such as age, sex, household income, education levels, material status, and prevalence of chronic diseases (e.g., hypertension, diabetes mellitus, hyperlipidemia, arthritis, and osteoporosis). Anthropometry was used to measure the weight, height, and body mass index. Household income was categorized into quartiles (low, middle-low, middle-high, and high). Education level was classified as elementary school, middle school, high school, or college graduate or higher. Marital status included married/living together, married/living separately, spouse died, divorced, and not married. Participants were identified as having diabetes when their fasting glucose level was ≥ 126 mg/dL, HbA1c ≥ 6.5%, or use of medication [25]. Hypertension was defined as systolic blood pressure (SBP) ≥ 140 mmHg, diastolic blood pressure (DBP) ≥ 90 mmHg, or using anti-hypertensive medications [25]. Hypertension was classified based on SBP ≥ 140 mmHg/DBP ≥ 90 mmHg or using anti-hypertensive medications [26]. Other chronic diseases were defined as having been diagnosed by a doctor or currently taking medicine.

### Data analysis

Participants’ characteristics were presented using descriptive analyses. To compare the differences in characteristics, we performed an independent *t*-test for continuous variables and a chi-squared (*χ*^2^-test) for categorical variables. Each domain-specific PA was categorized into two groups (no PA vs. any PA) based on the amount of PA. Based on the median value, sedentary behavior was divided into < 7 h/day and ≥ 7 h/day.

Multivariable-adjusted logistic regression was performed to estimate the odds ratios (ORs) and 95% confidence intervals (CIs) of the association with domain-specific PA, sedentary behavior, subjective health, and HR-QoL. Additionally, the joint association between domain-specific PA, sedentary behavior, subjective health, and HR-QoL was investigated using multiple-adjusted logistic regression. We included covariates such as age, sex, household income, education level, material status, and diseases to adjust for potential confounders. After adjusting for confounding factors, an analysis of covariance (ANCOVA) was performed to investigate the relationship between domain-specific PA, sedentary behavior, and the EQ-5D index.

To assess the robustness of our findings, we also conducted a subgroup analysis to explore whether the association of domain-specific PA, sedentary behavior, subjective health, and HR-QoL differed according to sociodemographic parameters. All analyses were conducted using SPSS software for Windows (version 26.0; IBM Corp., Armonk, NY), and statistical tests were 2-tailed, and *p* values < 0.05 were considered significant.

## Results

### Demographical characteristics of participants

Participants were grouped by sex; their characteristics are presented in Table 1. The mean age was 73.05 ± 5.12 years (male:72.91 ± 5.06 years, female:73.16 ± 5.17 years) and the body mass index was 24.09 ± 3.17 kg/m^2^ (male:23.76 ± 2.95 kg/m^2^, female:24.34 ± 3.31 kg/m^2^). Hypertension was the most common disease in both males (57%) and females (64%). Only 29.1% of the elderly met the aerobic PA guideline, and 17.9% were engaged in sufficient resistance exercise.

**Table 1:** Participants characteristics

		Total (n = 6,004)	Male (n = 2,598)	Female (n = 3,406)
Arthrometric marker				
	Age (years)	73.05 ± 5.12	72.91 ± 5.06	73.16 ± 5.17
	Weight (kg)	60.22 ± 1.11	65.41 ± 9.48	56.33 ± 8.73
	BMI (kg/m^2^)	24.09 ± 3.17	23.76 ± 2.95	24.34 ± 3.31
Education level (n,%)				
	≤ Middle school	4,241 (70.7)	1,456 (56.0)	2,785 (81.8)
	High school	1083 (18.0)	673 (25.9)	410 (12.0)
	≥ College	601 (10.0)	434 (16.7)	167 (4.9)
	No response	79 (1.3)	35 (1.4)	44 (1.3)
Marital status (n,%)				
	Married/live together	4,005 (66.7)	2,261 (87.0)	1,744 (51.2)
	Others ^a^	1,953 (32.6)	317 (12.2)	1,636 (48.0)
	No response	46 (0.7)	20 (0.8)	26 (0.8)
Income (n,%)				
	High	599 (10)	315 (12.1)	284 (8.3)
	Middle	2,624 (43.7)	1,261 (48.6)	1,363 (40.0)
	Low	2,749 (45.8)	1,011 (38.9)	1,738 (51.0)
	No response	32 (0.5)	11 (0.4)	21 (0.7)
Disease prevalence (n,%)				
	Hypertension	3,662 (61.0)	1481 (57.0)	2,181 (64.0)
	Diabetes mellites	841 (14.0)	400 (15.4)	441 (12.9)
	Hyperlipidemia	2,113 (35.2)	696 (26.8)	1,417 (41.6)
	Arthritis	1,759 (29.3)	346 (13.3)	1,413 (41.5)
	Osteoporosis	1,255 (20.9)	86 (3.3)	1,169 (34.3)
Meeting for PA guideline (n,%)				
	Aerobic PA	1,745 (29.1)	855 (32.9)	890 (26.1)
	Strengthening	1,079 (17.9)	485 (29.1)	322 (9.4)

Variables are presented as mean ± standard deviation (SD) or number (%); ^a^ Others include married/live separately, spouse died, divorce, and not married

### Association of domain-specific PA, subjective health, and HR-QoL

When we analyzed the sum of all domains’ PA, older persons who participated in PA showed a positive correlation with subjective health and HR-QoL parameters (Table 2). Individuals with any PA per week perceived themselves as healthier (OR = 1.36, 95% CI 1.18–1.58) and reported being less likely to have problems with HR-QoL parameters from 27% (OR = 0.73, 95% CI 0.63–0.86) to 40% (OR = 0.60, 95% CI 0.49–0.73) compared to those who did not do physical activity per week.

**Table 2:** The odds ratio of health-related quality of life according to domain-specific physical activity in Korean older adults

		Total PA		Leisure PA		Transportation PA		Work PA	
		No PA/week	Any PA/week	No PA/week	Any PA/week	No PA/week	Any PA/week	No PA/week	Any PA/week
**Healthy**									
	Crude	Ref	**1.59 (1.40–1.81)**	Ref	**2.40 (2.01–2.78)**	Ref	**1.35 (1.19–1.53)**	Ref	**0.64 (0.41–0.99)**
	Adjusted ^**a**^	Ref	**1.36 (1.18–1.58)**	Ref	**1.73 (1.44–2.07)**	Ref	**1.21 (1.05–1.39)**	Ref	**0.60 (0.37–0.97)**
	PA increase 60 min ^**a**^	**1.05 (1.03–1.08)**		**1.09 (1.05–1.13)**		**1.04 (1.01–1.07)**		0.93 (0.84–1.04)	
**Mobility problems**									
	Crude	Ref	**0.52 (0.47–0.58)**	Ref	**0.37 (0.30–0.44)**	Ref	**0.58 (0.52–0.64)**	Ref	**1.76 (1.28–2.41)**
	Adjusted ^**a**^	Ref	**0.69 (0.61–0.79)**	Ref	**0.59 (0.48–0.73)**	Ref	**0.71 (0.62–0.80)**	Ref	**2.17 (1.50–3.14)**
	PA increase 60 min ^**a**^	**0.92 (0.89–0.94)**		**0.90 (0.86–0.95)**		**0.92 (0.89–0.95)**		**1.12 (1.04–1.22)**	
**Self-care problems**									
	Crude	Ref	**0.41 (0.34–0.49)**	Ref	**0.31 (0.21–0.45)**	Ref	**0.44 (0.36–0.52)**	Ref	1.26 (0.77–2.05)
	Adjusted ^**a**^	Ref	**0.60 (0.49–0.73)**	Ref	**0.58 (0.38–0.86)**	Ref	**0.58 (0.47–0.72)**	Ref	1.47 (0.86–2.50)
	PA increase 60 min ^**a**^	**0.91 (0.86–0.95)**		**0.90 (0.81–0.99)**		**0.89 (0.84–0.94)**		1.03 (0.90–1.18)	
**Usual activity problems**									
	Crude	Ref	**0.51 (0.44–0.58)**	Ref	**0.39 (0.30–0.50)**	Ref	**0.54 (0.47–0.62)**	Ref	**1.72 (1.21–2.46)**
	Adjusted ^**a**^	Ref	**0.73 (0.63–0.86)**	Ref	**0.67 (0.50–0.89)**	Ref	**0.70 (0.60–0.82)**	Ref	**2.38 (1.59–3.56)**
	PA increase 60 min ^**a**^	**0.92 (0.89–0.96)**		**0.92 (0.85–0.98)**		**0.90 (0.86–0.94)**		**1.13 (1.03–1.24)**	
**Pain/discomfort**									
	Crude	Ref	**0.68 (0.61–0.75)**	Ref	**0.58 (0.49–0.69)**	Ref	**0.71 (0.64–0.79)**	Ref	**1.71 (1.25–2.36)**
	Adjusted ^**a**^	Ref	**0.82 (0.73–0.93)**	Ref	**0.78 (0.65–0.94)**	Ref	**0.83 (0.73–0.94)**	Ref	**1.96 (1.38–2.78)**
	PA increase 60 min ^**a**^	**0.95 (0.93–0.98)**		**0.95 (0.91–0.99)**		**0.95 (0.92–0.98)**		**1.10 (1.01–1.18)**	
**Anxiety/depressed**									
	Crude	Ref	**0.71 (0.62–0.83)**	Ref	**0.66 (0.52–0.84)**	Ref	**0.74 (0.64–0.86)**	Ref	1.28 (0.84–1.95)
	Adjusted ^**a**^	Ref	0.87 (0.74–1.03)	Ref	0.92 (0.71–1.19)	Ref	0.85 (0.72–1.01)	Ref	1.33 (0.84–2.11)
	PA increase 60 min ^**a**^	**0.95 (0.92–0.99)**		0.94 (0.88–1.01)		0.96 (0.92-1.00)		1.02 (0.92–1.14)	
**EQ-5D index** ^**a**^		0.87 ± 0.17	0.92 ± 0.12^***^	0.88 ± 0.16	0.94 ± 0.10^***^	0.86 ± 0.17	0.91 ± 0.12^***^	0.89 ± 0.15	0.84 ± 0.17^***^

Data represented as odds ratio (95% Confidence Interval: CI) or mean ± SD. Abbreviation: physical activity; PA. **BOLD** = *p* < 0.05, ^**a**^=adjust for age, gender, income, material status, education level, disease (hypertension, diabetes, hyperlipidemia, arthritis, osteoporosis), sedentary behavior

However, different relationships were observed in the analyses of domain-specific PA. Leisure time and transport PA were significantly positively correlated with subjective good health and HR-QoL. In contrast, work PA was significantly inversely associated with the odds of subjective good health and HR-QoL (Table 2). Individuals who participated in leisure or transportation PA were more likely to be identified as healthy by 73% (OR = 1.73, 95% CI 1.44–2.07) and 21% (OR = 1.21, 95% CI 1.05–1.39), respectively, compared with those who did not participate in these activities. In addition, for every 60 min per week of leisure- or transportation-related PA, the subjective health awareness OR increased by 9% (OR = 1.09, 95% CI 1.05–1.13) and 4% (OR = 1.04, 95% CI 1.01–1.07), respectively. Each leisure- and transportation-related activity also showed a dose-response relationship with the HR-QoL variables, as well as the EQ-5D index. A similar trend was observed when we conducted a sensitivity analysis by sex, age, BMI, education level, income status, and chronic disease status (Supplementary Table 1).

In contrast, older adults who engaged in work-related PA were 40% (OR = 0.60, 95% CI 0.37–0.97) were less likely to be identified as healthy than those with no work-related PA. In addition, individuals who have work-related PA were 1.33 times (OR = 1.33, 95% CI 0.84–2.11) to 2.38 times (OR = 2.38, 95% CI 1.59–3.56; *p* < 0.05) more likely to have problems with each HR-QoL dimensions compared to those who did not engage in work-related activity. The EQ-5D index was also significantly lower in older persons with work-related PA than in those without PA (0.84 ± 0.17 vs. 0.89 ± 0.15; *p* < 0.001).

### Association of sedentary behavior, subjective health, and HR-QoL

Sedentary time showed a significant negative association with the odds of subjectively positive health and HR-QoL parameters (Table 3). Compared to the older adults who spent < 7 h per day sedentary behavior (low sedentary), those who spent ≥ 7 h/day sedentary (high sedentary) were 14% (OR = 0.86, 95% CI 0.74–0.99) less likely to self-report as healthy. Furthermore, for every 60 min of increased sedentary behavior per day, the subjective health awareness OR decreased by 3% (OR = 0.97, 95% CI 0.94–0.99). In terms of the dose-response relationship, sedentary behavior declined in HR-QoL and EQ-5D index. Compared to individuals on a low sedentary trajectory, those who were highly sedentary reported higher odds of having mobility problems (OR = 1.44, 95% CI 1.26–1.65), self-care problems (OR = 1.47, 95% CI 1.17–1.86), usual care problems (OR = 1.83, 95% CI 1.53–2.20), and pain and discomfort (OR = 1.57, 95% CI 1.38–1.80). Particularly, highly sedentary individuals had a significantly lower EQ-5D index than those with low sedentary behavior (0.88 ± 0.16 vs. 0.92 ± 0.12; *p* < 0.001). These trends were observed in the subgroup analysis by sex, age, body mass index, education level, income level, and chronic disease status (Supplementary Table 1).

**Table 3:** The odds ratio of health-related quality of life according to sedentary behavior in Korean older adults

		Sedentary behavior	
		< 7 h/day	≥ 7 h/day
**Healthy**			
	Crude	Ref	**0.77 (0.68–0.88)**
	Adjusted ^**a**^	Ref	**0.86 (0.74–0.99)**
	sedentary increase 60 min ^**a**^	**0.97 (0.94–0.99)**	
**Mobility problems**			
	Crude	Ref	**1.75 (1.55–1.97)**
	Adjusted ^**a**^	Ref	**1.44 (1.26–1.65)**
	sedentary increase 60 min ^**a**^	**1.09 (1.07–1.12)**	
**Self-care problems**			
	Crude	Ref	**1.98 (1.59–2.45)**
	Adjusted ^**a**^	Ref	**1.47 (1.17–1.86)**
	sedentary increase 60 min ^**a**^	**1.11 (1.06–1.15)**	
**Usual activity problems**			
	Crude	Ref	**2.28 (1.93–2.69)**
	Adjusted ^**a**^	Ref	**1.83 (1.53–2.20)**
	sedentary increase 60 min ^**a**^	**1.14 (1.11–1.18)**	
**Pain/discomfort**			
	Crude	Ref	**1.72 (1.53–1.94)**
	Adjusted ^**a**^	Ref	**1.57 (1.38–1.80)**
	sedentary increase 60 min ^**a**^	**1.09 (1.07–1.12)**	
**Anxiety/depressed**			
	Crude	Ref	**1.53 (1.29–1.82)**
	Adjusted ^**a**^	Ref	**1.40 (1.16–1.69)**
	sedentary increase 60 min ^**a**^	**1.07 (1.03–1.10)**	
**EQ-5D index** ^**a**^		0.92 ± 0.12	0.88 ± 0.16^***^

Data represented as odds ratio (95% Confidence Interval; CI) or mean ± SD. **BOLD** = *p* < 0.05, ***p < 0.001 between groups. ^**a**^=adjust for age, gender, income, material status, education level, disease (hypertension, diabetes, Hyperlipidemia, arthritis, osteoporosis), and physical activity level

### Joint association between domain-specific PA, sedentary behavior with HR-QoL

**Fig. 2:**
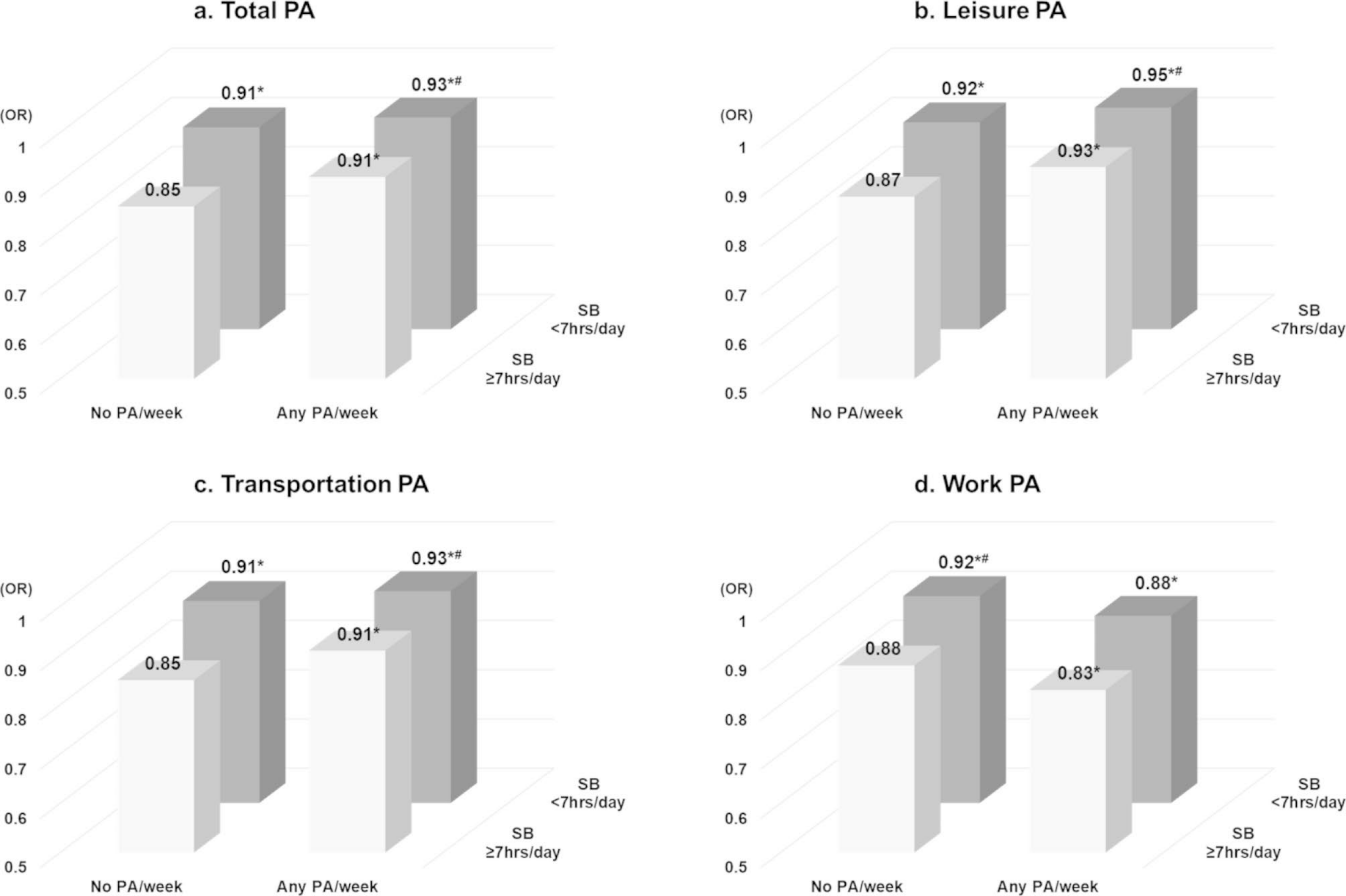
Joint associations between domain-specific physical activity and sedentary behavior with EQ5D index in Korean older adults Abbreviation: physical activity; PA, sedentary behavior; SB, *p < 0.05 with Sedentary behavior ≥ 7 h/day & No PA/week group, ^#^*p* < 0.05 with Sedentary behavior ≥ 7 h/day & any PA/week group. ^**a**^=adjust for age, gender, income, material status, education level, disease (hypertension, diabetes, hyperlipidemia, arthritis, osteoporosis)

The joint associations of domain-specific PA, sedentary behavior, subjective health, and HR-QoL are shown in Table 4; Fig. 2. When analyzing total PA, older people with more physically active or less sedentary behavior had higher odds of subjective good health and lower odds of having problems with HR-QoL variables. Additionally, older adults who were physically active and had less sitting time showed a significantly higher EQ-5D index compared to those who were physically inactive and had high sitting time (0.93 ± 0.11 vs. 0.85 ± 0.18; *p* < 0.05).

**Table 4:** Joint associations between domain-specific physical activity and sedentary behavior with health-related quality of life in Korean older adults

Total PA		Sedentary behavior ≥ 7hrs/day		Sedentary behavior < 7 hrs/day	
		No PA/week	Any PA/week	No PA/week	Any PA/week
Healthy					
	Crude	Ref	**1.71 (1.45–2.03)**	**1.44 (1.15–1.80)**	**1.93 (1.61–2.31)**
	Adjusted ^**a**^	Ref	**1.51 (1.26–1.81)**	**1.39 (1.09–1.77)**	**1.65 (1.35–2.01)**
**Mobility problems**					
	Crude	Ref	**0.53 (0.46–0.60)**	**0.54 (0.44–0.64)**	**0.36 (0.30–0.42)**
	Adjusted ^**a**^	Ref	**0.63 (0.54–0.74)**	**0.63 (0.51–0.78)**	**0.47 (0.39–0.56)**
**Self-care problems**					
	Crude	Ref	**0.39 (0.32–0.49)**	**0.46 (0.34–0.62)**	**0.28 (0.21–0.37)**
	Adjusted ^**a**^	Ref	**0.51 (0.40–0.65)**	**0.56 (0.40–0.78)**	**0.42 (0.31–0.57)**
**Usual activity problems**					
	Crude	Ref	**0.53 (0.45–0.62)**	**0.41 (0.32–0.53)**	**0.28 (0.23–0.35)**
	Adjusted ^**a**^	Ref	**0.67 (0.56–0.80)**	**0.49 (0.37–0.64)**	**0.39 (0.30–0.49)**
**Pain/discomfort**					
	Crude	Ref	**0.69 (0.60–0.79)**	**0.55 (0.46–0.67)**	**0.44 (0.38–0.52)**
	Adjusted ^**a**^	Ref	**0.79 (0.68–0.91)**	**0.61 (0.50–0.74)**	**0.51 (0.43–0.61)**
**Anxiety/depressed**					
	Crude	Ref	**0.69 (0.58–0.83)**	**0.52 (0.40–0.69)**	**0.56 (0.45–0.70)**
	Adjusted ^**a**^	Ref	**0.77 (0.64–0.94)**	**0.57 (0.42–0.76)**	**0.63 (0.50–0.80)**
**Leisure PA**		**Sedentary behavior ≥ 7hrs/day**		**Sedentary behavior < 7 hrs/day**	
		**No PA/week**	**Any PA/week**	**No PA/week**	**Any PA/week**
**Healthy**					
	Crude	Ref	**2.60 (2.10–3.20)**	**1.34 (1.15–1.56)**	**2.57 (2.00-3.30)**
	Adjusted ^**a**^	Ref	**1.96 (1.56–2.47)**	**1.29 (1.10–1.52)**	**1.88 (1.44–2.47)**
**Mobility problems**					
	Crude	Ref	**0.38 (0.30–0.48)**	**0.58 (0.51–0.66)**	**0.23 (0.17–0.32)**
	Adjusted ^**a**^	Ref	**0.60 (0.46–0.77)**	**0.67 (0.58–0.78)**	**0.36 (0.26–0.51)**
**Self-care problems**					
	Crude	Ref	**0.30 (0.19–0.48)**	**0.51 (0.40–0.63)**	**0.21 (0.11–0.42)**
	Adjusted ^**a**^	Ref	**0.53 (0.33–0.87)**	**0.63 (0.50–0.81)**	**0.38 (0.19–0.76)**
**Usual activity problems**					
	Crude	Ref	**0.40 (0.29–0.54)**	**0.44 (0.37–0.53)**	**0.20 (0.12–0.33)**
	Adjusted ^**a**^	Ref	**0.65 (0.47–0.91)**	**0.52 (0.43–0.63)**	**0.32 (0.19–0.53)**
**Pain/discomfort**					
	Crude	Ref	**0.62 (0.50–0.77)**	**0.59 (0.52–0.68)**	**0.34 (0.26–0.46)**
	Adjusted ^**a**^	Ref	0.83 (0.66–1.04)	**0.64 (0.56–0.74)**	**0.42 (0.31–0.57)**
**Anxiety/depressed**					
	Crude	Ref	**0.74 (0.55–0.99)**	**0.68 (0.56–0.81)**	**0.41 (0.26–0.64)**
	Adjusted ^**a**^	Ref	1.00 (0.73–1.37)	**0.72 (0.59–0.88)**	**0.52 (0.33–0.82)**
**Transportation PA**		**Sedentary behavior ≥ 7hrs/day**		**Sedentary behavior < 7 hrs/day**	
		**No PA/week**	**Any PA/week**	**No PA/week**	**Any PA/week**
**Healthy**					
	Crude	Ref	**1.42 (1.20–1.67)**	**1.39 (1.14–1.70)**	**1.62 (1.36–1.94)**
	Adjusted ^**a**^	Ref	**1.30 (1.09–1.55)**	**1.31 (1.05–1.62)**	**1.45 (1.19–1.76)**
**Mobility problems**					
	Crude	Ref	**0.58 (0.51–0.66)**	**0.53 (0.45–0.63)**	**0.39 (0.33–0.46)**
	Adjusted ^**a**^	Ref	**0.64 (0.55–0.75)**	**0.62 (0.52–0.76)**	**0.48 (0.40–0.57)**
**Self-care problems**					
	Crude	Ref	**0.43 (0.34–0.54)**	**0.48 (0.36–0.64)**	**0.29 (0.21–0.39)**
	Adjusted ^**a**^	Ref	**0.51 (0.40–0.66)**	**0.60 (0.44–0.82)**	**0.41 (0.30–0.57)**
**Usual activity problems**					
	Crude	Ref	**0.56 (0.47–0.66)**	**0.43 (0.34–0.54)**	**0.29 (0.23–0.36)**
	Adjusted ^**a**^	Ref	**0.65 (0.54–0.78)**	**0.51 (0.40–0.65)**	**0.37 (0.29–0.47)**
**Pain/discomfort**					
	Crude	Ref	**0.73 (0.63–0.83)**	**0.56 (0.47–0.67)**	**0.46 (0.39–0.55)**
	Adjusted ^**a**^	Ref	**0.79 (0.68–0.92)**	**0.61 (0.50–0.73)**	**0.51 (0.43–0.61)**
**Anxiety/depressed**					
	Crude	Ref	**0.69 (0.57–0.84)**	**0.51 (0.40–0.67)**	**0.60 (0.48–0.74)**
	Adjusted ^**a**^	Ref	**0.73 (0.60–0.89)**	**0.56 (0.42–0.73)**	**0.64 (0.50–0.81)**
**Work PA**		**Sedentary behavior ≥ 7hrs/day**		**Sedentary behavior < 7 hrs/day**	
		**No PA/week**	**Any PA/week**	**No PA/week**	**Any PA/week**
**Healthy**					
	Crude	Ref	0.64 (0.36–1.16)	**1.30 (1.14–1.49)**	0.75 (0.37–1.54)
	Adjusted ^**a**^	Ref	0.68 (0.37–1.27)	**1.24 (1.07–1.43)**	0.63 (0.30–1.34)
**Mobility problems**					
	Crude	Ref	**1.86 (1.23–2.80)**	**0.57 (0.50–0.65)**	1.06 (0.62–1.81)
	Adjusted ^**a**^	Ref	**2.06 (1.30–3.30)**	**0.65 (0.57–0.75)**	1.44 (0.80–2.61)
**Self-care problems**					
	Crude	Ref	0.95 (0.49–1.84)	**0.48 (0.39–0.60)**	1.30 (0.61–2.76)
	Adjusted ^**a**^	Ref	0.96 (0.48–1.91)	**0.60 (0.74–0.76)**	1.89 (0.85–4.18)
**Usual activity problems**					
	Crude	Ref	**1.72 (1.10–2.69)**	**0.43 (0.36–0.51)**	1.14 (0.61–2.13)
	Adjusted ^**a**^	Ref	**1.94 (1.19–3.14)**	**0.50 (0.41–0.60)**	1.61 (0.82–3.13)
**Pain/discomfort**					
	Crude	Ref	**1.51 (1.01–2.27)**	**0.57 (0.51–0.65)**	1.32 (0.78–2.33)
	Adjusted ^**a**^	Ref	**1.62 (1.04–2.51)**	**0.61 (0.53–0.69)**	1.57 (0.90–2.75)
**Anxiety/depressed**					
	Crude	Ref	1.34 (0.79–2.25)	**0.66 (0.55–0.78)**	0.79 (0.36–1.75)
	Adjusted ^**a**^	Ref	1.31 (0.76–2.27)	**0.69 (0.57–0.83)**	0.90 (0.40–2.05)
Data represented as odds ratio (95% Confidence Interval: CI). Abbreviation: physical activity; PA. **BOLD** = *p* < 0.05, *p < 0.05 with Sedentary behavior ≥ 7hrs/day & No PA/week group, ^#^*p* < 0.05 with Sedentary behavior ≥ 7hrs/day & any PA/week group. ^**a**^=adjust for age, gender, income, material status, education level, disease (hypertension, diabetes, hyperlipidemia, arthritis, osteoporosis).					

Data represented as odds ratio (95% Confidence Interval: CI). Abbreviation: physical activity; PA. **BOLD** = *p* < 0.05, *p < 0.05 with Sedentary behavior ≥ 7 h/day & No PA/week group, ^#^*p* < 0.05 with Sedentary behavior ≥ 7 h/day & any PA/week group. ^**a**^= adjust for age, gender, income, material status, education level, disease (hypertension, diabetes, hyperlipidemia, arthritis, osteoporosis)

However, subjective health and HR-QoL results according to domain-specific PA and sedentary time differed from total PA. In the case of leisure or transportation-related PA, individuals who were physically active or had less sitting time showed increased odds of subjectively good health and declined odds of responding to problems with HR-QoL components compared to those who had no PA or highly sedentary behavior. Compared to low sitting/active older adults, those who had high sitting time or were inactive in leisure or transportation PA significantly increased the odds of having problems in HR-QoL variables (Supplementary Table 2). Older adults who were physically active/have less sitting time also showed higher EQ-5D index than those who were the opposite (EQ-5D index in leisure 0.95 ± 0.09 vs. 0.87 ± 0.16; *p* < 0.05, in transportations 0.93 ± 0.11 vs. 0.85 ± 0.18; *p* < 0.05).

In contrast, work-related PA showed opposite results to leisure-or transportation-related PA. In terms of work-related PA, individuals who were physically active or had high sitting times tended to have lower odds of being healthy and higher odds of having HR-QoL problems. Even though sitting time was < 7 h/day, participants who did not perform work-related PA showed significantly lower odds of worse HR-QoL parameters than active individuals (Supplementary Table 2).

## Discussion

The main goal of the current study was to evaluate the association between domain-specific PA, sedentary behavior, subjective health perception, and HR-QoL in older adults. In our study, physically active older adults showed different associations with subjective health perception and HR-QoL, according to the PA domain. Additionally, older adults who engaged in sitting time or work-related PA showed an inverse association with subjective health perception and HR-QoL.

Unlike our results, Scarabottolo et al. [27] and Choi and Bum [28] reported that high activity in work or leisure PA benefitted functional capacity and general health perception. There are two main reasons for the difference between the results of prior studies and those of this study. First, this study used a different tool, the GPAQ, to measure PA levels. The amount of PA was calculated using the number of days and frequency of moderate and vigorous PA in each domain. However, in the case of Scarabottolo [27], work, sports, and leisure activities were divided into never, rarely, sometimes, often, and always. In addition, Choi’s [28] study was divided only by whether or not there was PA in work and leisure. In addition to the PA domain, the PA amount is closely related to quality-of-life parameters. Thus, additional research is needed to investigate the effects of quality of life and PA by measuring the exact amount of PA in each domain of the older population. Second, there are mixed meanings for PA at work. Work-related PA may imply both a physically healthy state in which one can continue working [29] and a state in which one has to work because of vulnerable sociodemographic characteristics [30–32]. Cunningham et al. [29] reported that PA levels in older adults are closely related to their activities of daily living (ADL). Therefore, older adults who continue working are more likely to be physically and potentially mentally healthy due to more social relationships. In contrast, Ryu et al. [30] reported an increase of 2.28 times the stress and depression in the case of moderate to vigorous PA at work. In particular, for every 100 metabolic equivalents (METs) increase in PA in simple blue-collar jobs, the risk of depression increased by 29% and 30% for men and women, respectively. Other jobs, such as white-collar jobs, showed no significant association between work-related PA and depression. In addition, the amount of PA associated with work is closely related to sociodemographic factors that may affect health and quality of life [31–33]. Therefore, it is necessary to understand these effects and consider the social and environmental factors affecting the quality of life when investigating the amount of work PA.

In this study, leisure time and transportation PA showed a positive correlation between subjectively good health and HR-QoL. Considering that transport-related PA is the most frequent domain of PA, follow-up research investigating the effect of transport-related PA is necessary [30, 34, 35]. PA is known to affect various determinant parameters such as income, level of education, and walking environment [32, 33, 36]. Thus, it is necessary to provide laws and systems to promote PA in older adults as social support.

Sedentary behavior was negatively correlated with mental health and HR-QoL. This study also confirmed that sedentary behavior was negatively associated with subjective health and HR-QoL perception. Additionally, we observed that older adults with high sitting time, who were also highly engaged in transportation or leisure PA, had less severe subjective health and HR-QoL scores. Tully et al. [37] supported these results in terms of sedentary behavior and PA’s relationship with physical and mental health.

The study’s limitations include: First, it was a cross-sectional study, making it difficult to explain the causal relationship between PA, sedentary behavior, perception of subjective health, and HR-QoL. Second, the study participants comprised Korean senior citizens aged 65 years or older; therefore, caution is required when compared with other races and cultures. Despite these limitations, this study is meaningful because it is the first to investigate the importance of domain-specific PA in older adults, and its relationship with sedentary behavior and HR-QoL using national data, which is a large sample size. In the future, a large-scale cohort study that considers various socio-environmental variables that affect the PA and HR-QoL of the elderly is needed. Also it is necessary to continuously examine policy support in order to improve older people’s HR-QoL, by first investigating inequality factors (within the elderly) from various angles.

## Conclusion

This study is the first to confirm the importance of reducing sedentary behavior, along with improving transportation and leisure-time related PA, to improve the HR-QoL of older adults. Considering the worldwide trend of aging populations, it is necessary to care for the physical and mental health of older adults. Continuous research on PA and reducing sedentary behavior is required to achieve this.

## Electronic supplementary material

Below is the link to the electronic supplementary material.

Supplementary Material 1

## Data Availability

Not applicable.

## Abbreviations

PA: Physical activity
HR-QoL: Health-related quality of life
