# Supplementary material for: Domain-specific physical activity, sedentary behavior, subjective health, and health-related quality of life among older adults
Source: Health Qual Life Outcomes. 2023 May 29;21:52. doi: 10.1186/s12955-023-02136-8 (PMC10228102; doi:10.1186/s12955-023-02136-8)
Supplement: Supplementary file 1 — Supplementary Material 1 [file 12955_2023_2136_MOESM1_ESM.docx]

**[supplementary material]**

**Table 1. Subgroup analysis between domain-specific physical activity and sedentary behavior with EQ5D index in Korean older adults**

| ***Total PA ^a^*** | | **No PA** | **Any PA** |
| --- | --- | --- | --- |
|  | Male | 0.90±0.16 | 0.93±0.11*** |
|  | Female | 0.83±0.19 | 0.90±0.13*** |
|  | <75 years | 0.89±0.16 | 0.92±0.11*** |
|  | Over 75 years | 0.82±0.19 | 0.89±0.13*** |
|  | BMI<23 | 0.87±0.17 | 0.92±0.12*** |
|  | BMI≥23 | 0.86±0.17 | 0.91±0.12*** |
|  | Education level=Low | 0.83±0.19 | 0.89±0.13*** |
|  | Education level= high | 0.90±0.15 | 0.94±0.10*** |
|  | Income level=Low | 0.83±0.19 | 0.89±0.14*** |
|  | Income level=High | 0.89±0.16 | 0.93±0.11*** |
|  | Chronic disease | 0.86±0.17 | 0.91±0.12* |
|  | Non-chronic disease | 0.92±0.12 | 0.94±0.10** |
| ***Leisure PA ^a^*** | | **No PA** | **Any PA** |
|  | Male | 0.91±0.14 | 0.95±0.08*** |
|  | Female | 0.86±0.17 | 0.91±0.12* |
|  | <75 years | 0.90±0.14 | 0.94±0.10** |
|  | Over 75 years | 0.85±.17 | 0.92±0.12* |
|  | BMI<23 | 0.89±0.15 | 0.95±0.10** |
|  | BMI≥23 | 0.88±0.15 | 0.93±0.10** |
|  | Education level= Low | 0.86±.17 | 0.90±0.13** |
|  | Education level= high | 0.91±0.13 | 0.95±0.09*** |
|  | Income level=Low | 0.85±0.17 | 0.91±0.13** |
|  | Income level=High | 0.91±0.14 | 0.95±0.09** |
|  | Chronic disease | 0.88±0.15 | 0.93±0.11* |
|  | Non-chronic disease | 0.93±0.11 | 0.96±0.07* |
| ***Transportation PA ^a^*** | | **No PA** | **Any PA** |
|  | Male | 0.90±0.15 | 0.93±0.11*** |
|  | Female | 0.83±0.19 | 0.90±0.13*** |
|  | <75 years | 0.89±0.15 | 0.92±0.11*** |
|  | Over 75 years | 0.82±0.19 | 0.89±0.13*** |
|  | BMI<23 | 0.87±0.17 | 0.92±0.12*** |
|  | BMI≥23 | 0.87±0.17 | 0.91±0.12*** |
|  | Education level=Low | 0.84±0.19 | 0.89±0.13*** |
|  | Education level= high | 0.91±0.14 | 0.94±0.10*** |
|  | Income level=Low | 0.83±0.19 | 0.89±0.13*** |
|  | Income level=High | 0.90±0.15 | 0.93±0.11*** |
|  | Chronic disease | 0.86±0.17 | 0.91±0.12* |
|  | Non-chronic disease | 0.93±0.12 | 0.94±0.09** |
| ***Work PA ^a^*** | |  |  |
|  | Male | 0.92±0.13 | 0.91±0.14 |
|  | Female | 0.87±0.16 | 0.78±0.17*** |
|  | <75 years | 0.91±0.13 | 0.87±0.16*** |
|  | Over 75 years | 0.86±0.17 | 0.79±0.18* |
|  | BMI<23 | 0.90±0.15 | 0.84±0.17* |
|  | BMI≥23 | 0.89±0.15 | 0.85±0.16*** |
|  | Education level= Low | 0.86±0.17 | 0.80±0.17*** |
|  | Education level= high | 0.92±0.12 | 0.89±0.16* |
|  | Income level=Low | 0.86±0.17 | 0.80±0.19** |
|  | Income level=High | 0.92±0.13 | 0.88±0.14** |
|  | Chronic disease | 0.89±0.15 | 0.84±0.17*** |
|  | Non-chronic disease | 0.94±0.10 | 0.89±0.17** |
| ***Sedentary behavior ^b^*** | | **≥7hrs/day** | **< 7 hrs/day** |
|  | Male | 0.94±0.10 | 0.91±0.14*** |
|  | Female | 0.91±0.12 | 0.86±0.17*** |
|  | <75 years | 0.93±0.11 | 0.90±0.14*** |
|  | Over 75 years | 0.90±0.13 | 0.84±0.17*** |
|  | BMI<23 | 0.92±0.12 | 0.88±0.15*** |
|  | BMI≥23 | 0.92±0.12 | 0.88±0.15*** |
|  | Education level=Low | 0.90±0.13 | 0.85±0.17*** |
|  | Education level= high | 0.94±0.10 | 0.91±0.13*** |
|  | Income level=Low | 0.90±0.12 | 0.84±0.17*** |
|  | Income level=High | 0.94±0.11 | 0.91±0.13*** |
|  | Chronic disease | 0.91±0.12 | 0.87±0.16* |
|  | Non-chronic disease | 0.96±0.08 | 0.92±0.12* |
| Data represented as mean±SD. ****p*<0.001, ***p*<0.01, **p*<0.05 between groups. ^a^= adjusted for sedentary behavior, ^b^=adjusted for physical activity levels, abbreviation: physical activity; PA, hours; hrs, chronic disease=elderly who with diabetes, hypertension, arthritis, osteoporosis | | | |

**Table 2. Joint associations between domain-specific physical activity and sedentary behavior with subjective health and health-related quality of life in Korean older adults**

| **Total PA** | | **Sedentary behavior ≥7hrs/day** | | **Sedentary behavior < 7 hrs/day** | |
| --- | --- | --- | --- | --- | --- |
|  |  | **No PA/week** | **Any PA/week** | **No PA/week** | **Any PA/week** |
| **Healthy** | |  |  |  |  |
|  | Crude | **0.52 (0.43-0.62)** | 0.89 (0.75-1.05) | **0.75 (0.60-0.94)** | Ref |
|  | Adjusted **^a^** | **0.61 (0.50-0.74)** | 0.92 (0.77-1.10) | 0.84 (0.67-1.07) | Ref |
| **Mobility problems** | |  |  |  |  |
|  | Crude | **2.82 (2.40-3.31)** | **1.48 (1.26-1.74)** | **1.51 (1.23-1.85)** | Ref |
|  | Adjusted **^a^** | **2.14 (1.79-2.56)** | **1.36 (1.4-1.62)** | **1.35 (1.08-1.70)** | Ref |
| **Self-care problems** | |  |  |  |  |
|  | Crude | **3.60 (2.70-4.80)** | **1.42 (1.04-1.94)** | **1.65 (1.13-2.40)** | Ref |
|  | Adjusted **^a^** | **2.37 (1.74-3.23)** | 1.22 (0.88-1.69) | 1.33 (0.89-1.99) | Ref |
| **Usual activity problems** | |  |  |  |  |
|  | Crude | **3.56 (2.85-4.44)** | **1.89 (1.50-2.37)** | **1.46 (1.08-1.96)** | Ref |
|  | Adjusted **^a^** | **2.60 (2.04-3.30)** | **1.73 (1.35-2.22)** | 1.27 (0.92-1.74) | Ref |
| **Pain/discomfort** | |  |  |  |  |
|  | Crude | **2.25 (1.92-2.64)** | **1.55 (1.32-1.82)** | **1.25 (1.02-1.54)** | Ref |
|  | Adjusted **^a^** | **1.95 (1.64-2.32)** | **1.53 (1.29-1.82)** | 1.19 (0.95-1.48) | Ref |
| **Anxiety/depressed** | |  |  |  |  |
|  | Crude | **1.79 (1.44-2.22)** | 1.24 (0.99-1.55) | 0.94 (0.69-1.27) | Ref |
|  | Adjusted **^a^** | **1.59 (1.26-2.02**) | 1.23 (0.97-1.56) | 0.91 (0.66-1.25) | Ref |
| **Leisure PA** | | **Sedentary behavior ≥7hrs/day** | | **Sedentary behavior < 7 hrs/day** | |
|  |  | **No PA/week** | **Any PA/week** | **No PA/week** | **Any PA/week** |
| **Healthy** | |  |  |  |  |
|  | Crude | **0.39 (0.30-0.50)** | 1.01 (0.75-1.36) | **0.52 (0.40-0.68)** | Ref |
|  | Adjusted **^a^** | **0.53 (0.41-0.70)** | 1.04 (0.76-1.43) | **0.69 (0.52-0.91)** | Ref |
| **Mobility problems** | |  |  |  |  |
|  | Crude | **4.31 (3.12-5.96)** | **1.63 (1.11-2.39)** | **2.49 (1.78-3.48)** | Ref |
|  | Adjusted **^a^** | **2.76 (1.96-3.89)** | **1.65 (1.10-2.48)** | **1.85 (1.30-2.64)** | Ref |
| **Self-care problems** | |  |  |  |  |
|  | Crude | **4.71 (2.40-9.21)** | 1.41 (0.63-3.15) | **2.38 (1.19-4.75)** | Ref |
|  | Adjusted **^a^** | **2.62 (1.32-5.20)** | 1.39 (0.62-3.15) | 1.66 (0.82-3.36) | Ref |
| **Usual activity problems** | |  |  |  |  |
|  | Crude | **5.02 (3.06-8.25)** | **2.00 (1.13-3.54)** | **2.22 (1.33-3.70)** | Ref |
|  | Adjusted **^a^** | **3.13 (1.88-5.20)** | **2.04 (1.14-3.66)** | 1.63 (0.96-2.75) | Ref |
| **Pain/discomfort** | |  |  |  |  |
|  | Crude | **2.92 (2.18-3.91)** | **1.82 (1.29-2.56)** | **1.74 (1.28-2.35)** | Ref |
|  | Adjusted **^a^** | **2.39 (1.75-3.26)** | **1.98 (1.38-2.85)** | **1.53 (1.11-2.11)** | Ref |
| **Anxiety/depressed** | |  |  |  |  |
|  | Crude | **2.45 (1.57-3.81)** | **1.80 (1.08-3.01)** | **1.65 (1.04-2.62)** | Ref |
|  | Adjusted **^a^** | **1.92 (1.22-3.03)** | **1.92 (1.14-3.24)** | 1.38 (0.86-2.21) | Ref |
| **Transportation PA** | | **Sedentary behavior ≥7hrs/day** | | **Sedentary behavior < 7 hrs/day** | |
|  |  | **No PA/week** | **Any PA/week** | **No PA/week** | **Any PA/week** |
| **Healthy** | |  |  |  |  |
|  | Crude | **0.62 (0.52-0.74)** | 0.87 (0.73-1.04) | 0.86 (0.69-1.06) | Ref |
|  | Adjusted **^a^** | **0.69 (0.57-0.84**) | 0.90 (0.74-1.09) | 0.90 (0.72-1.13) | Ref |
| **Mobility problems** | |  |  |  |  |
|  | Crude | **2.55 (2.16-3.00)** | **1.48 (1.24-1.75)** | **1.36 (1.11-1.66)** | Ref |
|  | Adjusted **^a^** | **2.09 (1.74-2.51)** | **1.34 (1.11-1.62)** | **1.31 (1.04-1.63)** | Ref |
| **Self-care problems** | |  |  |  |  |
|  | Crude | **3.50 (2.58-4.74)** | **1.49 (1.06-2.09)** | **1.69 (1.16-2.47)** | Ref |
|  | Adjusted **^a^** | **2.45 (1.77-3.39)** | 1.26 (0.88-1.80) | 1.47 (0.99-2.20) | Ref |
| **Usual activity problems** | |  |  |  |  |
|  | Crude | **3.48 (2.76-4.39)** | **1.93 (1.51-2.48)** | **1.48 (1.10-1.99)** | Ref |
|  | Adjusted **^a^** | **2.73 (2.12-3.51)** | **1.76 (1.35-2.30)** | **1.39 (1.01-1.90)** | Ref |
| **Pain/discomfort** | |  |  |  |  |
|  | Crude | **2.16 (1.84-2.54)** | **1.57 (1.32-1.86)** | 1.21 (0.99-1.48) | Ref |
|  | Adjusted **^a^** | **1.94 (1.63-2.32)** | **1.53 (1.28-1.84)** | 1.18 (0.95-1.46) | Ref |
| **Anxiety/depressed** | |  |  |  |  |
|  | Crude | **1.68 (1.35-2.10)** | 1.16 (0.92-1.48) | 0.86 (0.64-1.16) | Ref |
|  | Adjusted **^a^** | **1.57 (1.24-1.99)** | 1.15 (0.89-1.48) | 0.87 (0.64-1.20) | Ref |
| **Work PA** | | **Sedentary behavior ≥7hrs/day** | | **Sedentary behavior < 7 hrs/day** | |
|  |  | **No PA/week** | **Any PA/week** | **No PA/week** | **Any PA/week** |
| **Healthy** | |  |  |  |  |
|  | Crude | 1.33 (0.65-2.73) | 0.86 (0.34-2.15) | 1.73 (0.84-3.55) | Ref |
|  | Adjusted **^a^** | 1.58 (0.75-3.33) | 1.07 (0.41-2.82) | 1.95 (0.92-4.13) | Ref |
| **Mobility problems** | |  |  |  |  |
|  | Crude | 0.94 (0.55-1.61) | 1.75 (0.90-3.42) | **0.54 (0.31-0.92)** | Ref |
|  | Adjusted **^a^** | 0.69 (0.38-1.25) | 1.43 (0.68-3.02) | **0.45 (0.25-0.82)** | Ref |
| **Self-care problems** | |  |  |  |  |
|  | Crude | 0.77 (0.36-1.64) | 0.73 (0.27-1.97) | **0.37 (0.17-0.81)** | Ref |
|  | Adjusted **^a^** | 0.53 (0.24-1.17) | 0.51 (0.18-1.43) | **0.32 (0.14-0.72)** | Ref |
| **Usual activity problems** | |  |  |  |  |
|  | Crude | 0.88 (0.47-1.64) | 1.51 (0.71-3.22) | **0.38 (0.20-0.71)** | Ref |
|  | Adjusted **^a^** | 0.62 (0.32-1.21) | 1.21 (0.53-2.72) | **0.31 (0.16-0.61)** | Ref |
| **Pain/discomfort** | |  |  |  |  |
|  | Crude | 0.76 (0.45-1.28) | 1.14 (0.59-2.21) | **0.43 (0.26-0.74)** | Ref |
|  | Adjusted **^a^** | 0.64 (0.36-1.11) | 1.03 (0.51-2.08) | **0.38 (0.22-0.68)** | Ref |
| **Anxiety/depressed** | |  |  |  |  |
|  | Crude | 1.27 (0.57-2.81) | 1.69 (0.66-4.35) | 0.83 (0.37-1.86) | Ref |
|  | Adjusted **^a^** | 1.11 (0.49-2.51) | 1.45 (0.55-3.86) | 0.76 (0.33-1.74) | Ref |
| Data represented as odds ratio (95% Confidence Interval; CI) or mean±SD. Abbreviation: physical activity; PA. **BOLD**=*p*<0.05, **p*<0.05 with Sedentary behavior ≥7hrs/day & No PA/week group, ^#^*p*<0.05 with Sedentary behavior ≥7hrs/day & any PA/week group. **^a^**=adjust for age, gender, income, material status, education level, disease (hypertension, diabetes, Hyperlipidemia, arthritis, osteoporosis). | | | | | |
